# Secondary Wall Regulating NACs Differentially Bind at the Promoter at a *CELLULOSE SYNTHASE A4 Cis*-eQTL

**DOI:** 10.3389/fpls.2018.01895

**Published:** 2018-12-21

**Authors:** Jennifer R. Olins, Li Lin, Scott J. Lee, Gina M. Trabucco, Kirk J.-M. MacKinnon, Samuel P. Hazen

**Affiliations:** ^1^Biology Department, University of Massachusetts, Amherst, MA, United States; ^2^Plant Biology Graduate Program, University of Massachusetts, Amherst, MA, United States; ^3^Molecular and Cellular Biology Graduate Program, University of Massachusetts, Amherst, MA, United States

**Keywords:** CELLULOSE SYNTHASE A4, NAC transcription factor, expression QTL, VNS element, tracheary element-regulating *cis*-element

## Abstract

*Arabidopsis thaliana* CELLULOSE SYNTHASE A4/7/8 (CESA4/7/8) are three non-redundant subunits of the secondary cell wall cellulose synthase complex. Transcript abundance of these genes can vary among genotypes and expression quantitative trait loci (eQTL) were identified in a recombinant population of the accessions Bay-0 and Shahdara. Genetic mapping and analysis of the transcript levels of *CESAs* between two distinct near isogenic lines (NILs) confirmed a change in *CESA4* expression that segregates within that interval. We sequenced the promoters and identified 16 polymorphisms differentiating *CESA4^Sha^* and *CESA4^Bay^*. In order to determine which of these SNPs could be responsible for this eQTL, we screened for transcription factor protein affinity with promoter fragments of *CESA4^Bay^, CESA4^Sha^*, and the reference genome *CESA4^Col^*. The wall thickening activator proteins NAC SECONDARY WALL THICKENING PROMOTING FACTOR2 (NST2) and NST3 exhibited a decrease in binding with the *CESA4^Sha^* promoter with a tracheary element-regulating *cis*-element (TERE) polymorphism. While NILs harboring the TERE polymorphisms exhibited significantly different *CESA4* expression, cellulose crystallinity and cell wall thickness were indistinguishable. These results suggest that the TERE polymorphism resulted in differential transcription factor binding and *CESA4* expression; yet *A. thaliana* is able to tolerate this transcriptional variability without compromising the structural elements of the plant, providing insight into the elasticity of gene regulation as it pertains to cell wall biosynthesis and regulation. We also explored available DNA affinity purification sequencing data to resolve a core binding site, C(G/T)TNNNNNNNA(A/C)G, for secondary wall NACs referred to as the VNS element.

## Introduction

While a primary cell wall surrounds all plant cells, a secondary cell wall is also found in xylem cells responsible for water transportation, structural fibers, and cells that serve as an outside barrier to the external environment. These thick and relatively inflexible walls are composed of a complex of cellulose, hemicelluloses, and the polyphenolic polymer lignin. Cellulose is the most abundant fraction in the majority of tissues and exists as long unbranched β-1,4-linked glucan chains. Cellulose chains coalesce in parallel to form a single microfibril via hydrogen bonding and van der Waals forces. Depending on their density and nature of the commingling polymers and linkages, microfibrils can contribute to a matrix that ranges from fairly elastic to extremely rigid. Aside from the obvious functional virtues to plants, secondary cell wall rich in cellulose are a valuable feedstock for the pulp and paper and the biofuel industries (Carroll and Somerville, 2009). Understanding the regulation of secondary cell wall composition, especially the effects of natural genetic variation, will facilitate enhanced gene modification and plant breeding for more efficient biomass production.

Cellulose is synthesized at the plasma membrane by multiple Cellulose Synthase A (CESA) proteins organized into rosette shaped complexes (Mueller and Brown, 1980). The rosette is composed of at least 18 CESA subunits organized into six globules, termed cellulose synthase complexes (CSCs) (Jarvis, 2013). There are ten proteins in the *A. thaliana* CESA family, while direct polymerization activity remains to be documented, all but CESA10 have been shown to be associated with cellulose biosynthesis (Guerriero et al., 2010). Mutations in a number of non-CESA genes, including members of the COBRA family, also exhibit polymerization defects (Ching et al., 2006; Sindhu et al., 2007; Zhang et al., 2010; Kotake et al., 2011). All members of the CESA superfamily, which includes the CESAs and seven CESA-like (CSL) families in eudicots, are integral membrane proteins; CESAs have two transmembrane domains in the N-terminus and six at the C-terminus, while CSLs are more variable (Somerville, 2006; Nixon et al., 2016; Little et al., 2018). Additionally, all ten CESA family members contain a LIM-like Zn-binding domain/RING finger, which is known to be involved in protein–protein interactions (Somerville, 2006). These attributes are in line with the finding that CESAs form a complex embedded in the cell membrane (Arioli et al., 1998; Fagard et al., 2000; Scheible et al., 2001; Taylor et al., 2003; Timmers et al., 2009). Across plant species and under most circumstances, three distinct non-redundant CESAs are required for optimal production of cellulose, and in plants, CESA1, 3, and one or more of 2/5/6/9 are involved in primary cell wall development, while CESA4, 7, and 8 are responsible for secondary cell wall biosynthesis (Kumar and Turner, 2015).

Early studies of knock-out mutations in CESA genes in *A. thaliana* first revealed the non-redundant nature of the secondary *CESA*s, as mutants harboring null mutations in any of the three secondary CESAs exhibited irregular cell walls and weaker stems (Taylor et al., 2000). To further understand the intricacies of the *CESA* genes, subsequent studies investigated the effects of more subtle changes in *CESA* expression. Virus-induced gene silencing (VIGS) of *CESA* genes in *Nicotiana benthamiana* led to dwarfed phenotypes and reduced cellulose content (Burton et al., 2000). A similar VIGS study in flax again resulted in plants of shorter stature that only displayed a slight reduction in sugar content (Chantreau et al., 2015). Comparable phenotypes were also observed in *Brachypodium distachyon* with compromised expression of *CESA* genes by use of artificial microRNAs, and more recently, it was shown that both *CESA* knock-down and overexpression induced similar biomass-compromised phenotypes in *Panicum virgatum L.* (Handakumbura et al., 2013; Mazarei et al., 2018).

Non-redundancy within secondary cell wall CESAs suggests the sensitive nature of cellulose synthesis and cell wall growth. In conjunction, the 18 *CESAs* in each rosette likely synthesize an individual β-1-4-glucan cellulose chain that coalesce in parallel to form a single microfibril via hydrogen bonding and van der Waals forces, though cases of 24-chain fibrils have been reported (Thomas et al., 2013). Variation among species in the orientation and size of the CSCs correlates with the size and thickness of microfibrils. Though the microfibrils have an organized, crystalline structure, the inner chains in the bundle tend to exhibit higher degrees of crystallinity, while sheath fibers are more disordered (Nixon et al., 2016). The degree of crystallinity within the microfibril has been found to be inversely correlated with the rate of polymerization. Moreover, disruptions to CESA domains that either encourage microfibril aggregation or membrane complex subunit associations show an overall reduction in crystallinity (Harris et al., 2012).

The expression of *CESA* genes and many others that encode additional cell wall components are highly co-regulated (Brown et al., 2005; Persson et al., 2005). A current model for transcriptional regulation of cell wall genes is a series of feed forward loops (Taylor-Teeples et al., 2015; Zhang et al., 2018). Transcription factors most commonly bind to the promoters of secondary cell wall genes associated with different classes of wall components as well as the promoters of other wall regulating transcription factors. Three phylogenetically distinct groups of NACs play the role of direct and indirect activators of cell wall gene expression: VNDs (VASCULAR-RELATED NAC-DOMAIN), NSTs (NAC SECONDARY WALL THICKENING), and SNDs (SECONDARY WALL-ASSOCIATED NAC DOMAIN PROTEIN) (Yao et al., 2012; Nakano et al., 2015). There are seven *A. thaliana* VNDs and all can activate xylem vessel cell differentiation (Kubo et al., 2005). Similarly, the NSTs positively activate cell wall thickening, but in fiber cells rather than vasculature (Mitsuda et al., 2007; Zhong et al., 2010). In *A. thaliana*, these include NST1, NST2, and NST3/SND1. The third clade, SNDs, consists of two *A. thaliana* genes, SND2 and SND3. Similar to the NSTs, SND2 and its orthologs in poplar, rice, and switchgrass can also directly and indirectly activate secondary cell wall thickening (Zhong et al., 2008; Hussey et al., 2011; Rao and Dixon, 2018; Ye et al., 2018). While these three classes of NAC transcription factors are distinct in their amino acid sequence as well as their role in wall development, they are reported to bind similar DNA sequences. The consensus sequence CTTNAAAGCNA, named the tracheary element-regulating cis-element (TERE), was initially identified in the promoters of genes associated with secondary cell wall formation and programmed cell death of vasculature (Pyo et al., 2007). Subsequently, VNDs, NSTs, and SNDs have been shown to bind directly and specifically to the TERE motif and a similar target, the secondary wall NAC-binding element [SNBE, (T/A)NN(C/T)(T/C/G)TNNNNNNNA(A/C)GN(A/C/T)(A/T)] (Pyo et al., 2007; Ohashi-Ito et al., 2010; Zhong et al., 2010; Taylor-Teeples et al., 2015; Ye et al., 2018). Thus, these NACs have both overlapping and distinct roles in the regulation of cell differentiation and secondary cell wall biosynthesis.

Naturally occurring genetic diversity offers a rich source from which to identify promising genes and variants for bioenergy crop breeding. Identification of markers associated with advantageous biomass accumulation traits has been carried out in chickpea, maize, sorghum, and other species, and such studies frequently pinpoint loci of *CESA* genes as potential targets (Shiringani and Friedt, 2011; Barrière et al., 2012; Zhao et al., 2013; Kujur et al., 2015). In addition, analysis of variable expression profiles among accessions has also been highlighted as a plant breeding tool, as exhibited by studies in sugarcane, loblolly pine, and shrub willow (Serapiglia et al., 2012; Palle et al., 2013; Kasirajan et al., 2018). Such studies contribute to the identification of candidate biomass accumulation genes, and also present a method of early selection in the breeding process. In general, transcriptome analysis suggests upregulation of *CESA* genes correlates with increased biomass (Serapiglia et al., 2012; Kujur et al., 2015; Kasirajan et al., 2018). In the present study, we investigated the causes and consequences of an eQTL at the *CESA4 cis*-eQTL in the *A. thaliana* Bayreuth (Bay-0) and Shakdara (Sha) recombinant inbred line (RIL) population (Loudet et al., 2002; West et al., 2007). We aimed to take advantage of the natural variation of *A. thaliana* to better understand the genotypic and phenotypic diversity for biofuel-relevant traits, specifically by studying the regulation of and variation in crystalline cellulose content within the plant cell wall.

## Materials and Methods

### Yeast One-Hybrid Protein-DNA Interaction Assays

Yeast one-hybrid protein–DNA interaction assays were conducted as previously described (Taylor-Teeples et al., 2015). The transcription factors were transformed into each yeast strain and the β-galactosidase activity was determined as previously described (Pruneda-Paz et al., 2009). Positive interactions were visually identified as incidence of yellow caused by the presence of ortho-nitrophenyl cleavage from colorless ortho-nitrophenyl-β-D-galactoside by β-galactosidase. The DNA bait strains were, similarly, tested for self-activation prior to screening, under selection but in the absence of any prey vector. A total of 34 *E. coli* strains harboring different *A. thaliana* transcription factors (Supplementary Table 1) were arrayed in 96-well plates and plasmids were prepared. proCESA4^Col^ (539 bp), proCESA4^Bay^ (539 bp), and proCESA4^Sha^ (540 bp) were cloned and recombined with reporter genes. Promoter sequences and primers used are described in Supplementary Table 2. Nine of overlapping fragments of CESA4^Col^ were independently cloned according to Pruneda-Paz et al. (2009). The oligonucleotides used to amplify promoter fragments are described in Supplementary Table 2. The screen was replicated in full to confirm the results and each clone was sequenced to re-confirm identity.

### Electrophoretic Mobility Shift Assays

To express recombinant NST2 or SND1 protein, coding sequences were cloned and fused to glutathione S-transferase tag in the pDONR211 vector and then transferred into pDEST15 (Invitrogen). *E. coli* strain BL21-AI (Invitrogen) transformed with pDEST15-GST:NST2 were grown in liquid media to an OD600 of 0.4, treated with 0.2% L-arabinose to induce expression overnight and harvested by centrifugation the following day. Cells were treated with 1 mg/mL lysozyme on ice for 30 min in minimal volume of 1X PBS buffer and lysed by sonication. Cell lysates were clarified by centrifugation and incubated with 100 μL of glutathione sepharose beads (GE Healthcare, Pittsburg, PA, United States) for 30 min at 4°C with rotation. The beads were transferred to a column, washed with 10 volumes of 1X PBS. Protein was eluted in 100 mM Tris-HCl pH8.0, 100 mM NaCl and 3 mg/mL glutathione buffer and purified protein was re-suspended in 50% glycerol and stored at -80°C.

Three overlapping probes were generated for *CESA4^Bay^-C, CESA4^Bay^-D, CESA4^Bay^-E, CESA4^Sha^-C, CESA4^Sha^-D, CESA4^Sha^-E* promoter fragments using the same oligonucleotides described in Supplementary Table 2. Reactions were carried out in binding buffer (10 mM Tris, pH7.5, 50 mM KCl, 1 mM DTT, 2.5% glycerol, 5 mM MgCl2, 0.1% IGEPAL CA-630, and 0.05 μg/μl calf thymus DNA). Following the addition of 150 ng of protein from the GST purification eluate, reactions were incubated at room temperature for 30 min. Protein-DNA complexes were separated from the free DNA on 1% agarose/1X TAE gels at 4°C. The agarose gels were stained with ethidium bromide and bands visualized under UV light.

### Characterization of Near Isogenic Lines

To develop NILs, RILs maintaining heterozygosity at the *CESA4* locus in the F6 generation were identified (Loudet et al., 2002). One plant per RIL carrying a heterozygous *CESA4* locus was identified via genotyping and selfed to obtain the F7 seeds. Within the F7 plants, pairs of lines were identified that were either homozygous for the *CESA4^Bay^* and *CESA4*^S^*^ha^* alleles to generate the NILs used in this study. The nearly isogenic lines analyzed in this study were developed from RIL93 and RIL350 segregating for the *CESA4* region. Individual HIF plants were genotyped for the Bay-0 or Sha *CESA4* allele by PCR of a 543 bp of CESA4 promoter with primers described in Supplementary Table 2. PCR products were subjected to restriction enzyme digestion with BsrDI (New England BioLabs, Ipswich, MA, United States) at 65°C for 2 h. The Sha allele incudes a BsRDI cut site (CGTTAC| NN) resulting in 401 and 142 bp fragments. The 543 bp Bay-0 allele contains no BrsDI restriction sites and remains undigested. Transcript abundance of *CESA4* in the near isogenic lines (NILs) was quantified in 5 cm stems. Stem tissue was frozen in liquid nitrogen and pulverized with metal beads in a Retsch (Haan, Germany) Mixer Mill MM400. RNA extraction and cDNA synthesis was conducted as described above. Primers for *CESA4* real-time PCR are described in Supplementary Table 2.

### Quantification of Crystalline Cellulose

To quantify and compare levels of crystalline cellulose, the Updegraff assay as adapted and described by Kumar and Turner was used (Updegraff, 1969; Kumar and Turner, 2015). Briefly, alcohol-insoluble residue (AIR) samples were first prepared from senesced stem tissue; samples were weighed at this stage for later calculations. Then, 3 mL of an acetic/nitric acid solution (8:1:2, acetic acid: nitric acid: water) was added to AIR samples and incubated in a boiling water bath for 30 min. This step removes hemicellulose and lignin while leaving cellulose microfibrils intact. The remaining cellulosic material was then swelled in 67% sulfuric acid in a boiling water bath for 5 min to disorganize the polymers, and the subsequent monomers were finally analyzed with a sulfuric acid/anthrone colorimetric assay. Absorbance was measured at a wavelength of 620 nm with a *SpectraMax M5* and corrected to a known glucose standard to calculate the percent cell wall composition of cellulose.

### Quantification of Stem Thickness

To investigate the histological effects of expression variation on the cell wall, 100 μm cross sections were sliced using a *Leica Biosystems* VT1000S Vibrating-blade microtome. Sections were incubated on the bench top for 2 min in a 2% w/v phloroglucinol/ethanol solution (Fisher Scientific, Waltham, MA), mounted in a 1:1 concentrated hydrochloric acid: water solution, and immediately imaged using a *Nikon* Eclipse E200 microscope and a *PixeLink* scope camera. For each stem, three sections were imaged, and five cells from three different portions of each stem were measured to get an average of 45 measurements per biological sample.

### Polarized Light Microscopy

To qualitatively analyze cellulose crystallinity under polarized light, internode segments were first cut on a vibratome into 100 μm sections, fixed in 2% glutaraldehyde and then embedded in Epon/Araldite (Sigma-Aldrich, St. Louis, MO, United States) before slicing into 0.5 μm sections on an Reichert-Jung, Ultracut E microtome (Vienna, Austria). Polarized light microscopy takes advantage of variation in the passage of light through crystalline structures to uncover discrete differences in crystallinity configurations. Specifically, the LC-PolScope (CRI, Cambridge, MA, United States) employed for this assay uses polarized light to measure birefringent retardance and the intensity of the images generated directly correlates to the retardance value, thus qualitative differences in crystallinity can be observed. Several stem samples from each HIF were imaged and analyzed.

### DNA Affinity Purification Sequence Analysis

In order to determine a consensus VND, NST, and SND (VNS) protein binding motif, the MEME.txt motif files from O’Malley et al. (2016) were visually aligned based on nucleotide similarity and trimmed to a length of 13 bases. An average for each nucleotide, at each motif position was calculated using a bash script that referenced the aligned and trimmed motif files. The consensus matrix was then loaded into R and the Bioconductor package SeqLogo was used to generate the motif logo. A bash shell script counted each of the VNS motif variant across the *A. thaliana* TAIR 10 sequence assembly and for each DAP-seq set of binding sites. Sum of the counts determined the total number of VNS motif sites and percentages were calculated as the proportion of each motif variant relative to the sum and multiplied by 100. The process was repeated for the DAP-seq binding site data using the reference genome file as a guide to extract DAP-seq peak fasta sequences. ThenarrowPeak DAP-seq peak data files were then searched iteratively through each of the peak binding sites. The percentage shown for DAP-seq data represents the mean percentage across all binding site files.

### Statistical Analysis

Correlation coefficients and tests of significance were calculated using Pearson’s correlation tests for all *CESA* genes pairwise using replicate gene expression data for Bay-0 and Sha parental lines and RILs using the rcorr function from the Hmisc library in R 3.2.5. Two tailed Student’s *t*-tests were carried out in Excel to assess gene expression, cell thickness, and cellulose content data sets. BoxPlots were generated with the BoxPlotR Web Tool (Spitzer et al., 2014).

### Accession Numbers

CESA1 (At4g32410), CESA2 (At4g39350), CESA3 (At5g05170), CESA4 (At5g44030), CESA5 (At5g09870), CESA6 (At5g64740), CESA7 (At5g17420), CESA8 (At4g18780), CESA9 (At2g21770), CESA10 (At2g25540), NST1 (At2g46770), NST2 (At3g61910), NST3/SND1 (At1g32770), PP2AA3 (At1g13320), TIP41 (At4g34270).

## Results

### Natural Variation and Co-expression of the Secondary Wall CESAs

**FIGURE 1:**
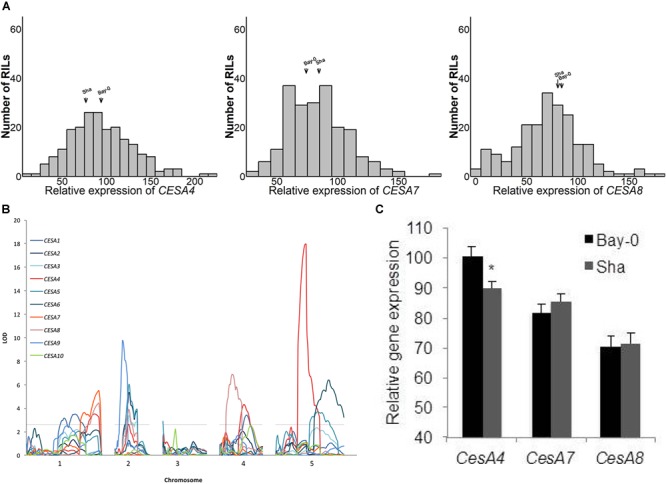
**(A)** Histograms of relative expression of *CESA4/7/8* within the Bay-0 × Sha recombinant inbred line population (RIL). **(B)** Genetic mapping identified a *CESA4* expression quantitative trait locus coincident with the physical position of the *CESA4* locus on chromosome five. **(C)** Relative expression of *CESA4/7/8* from RIL parents carrying Bay-0 or Sha allele. Lines carrying Bay-0 allele had significantly higher expression of *CesA4*, while no differences were observed between RILS for the expression of *CESA7* or *CESA8*. ^∗^*P* < 0.01.

We explored the observation that *CESA* gene expression varied among different accessions of *A. thaliana*. West et al. (2007) measured the expression of 22,746 genes in replicated samples of the Bay-0/Sha RIL population. Above ground tissue of 211 short-day grown plants was assayed after 6 weeks of growth using the Affymetrix ATH1 GeneChip microarray. There was a continuous range of values and normal distributions were observed for the ten *CESA* genes known to play a role in the biosynthesis of cellulose in secondary cell walls (Figure 1A and Supplementary Figure 1). The midparent values (i.e., the values halfway between the two parents) and the median of the RILs for *CESA4, CESA7*, and *CESA8* (Figure 1A) and the seven other *CESA* genes (Supplementary Figure 1) were very similar.

The *CESA* genes have been shown to be highly co-regulated within their functional classes. Three of the primary *CESA*s, *CESA1, 3*, and *6*, had the most similar gene expression to each other in a meta analysis of gene expression and the same is true among the secondary *CESA*s: *CESA4, 7*, and *8* (Persson et al., 2005). Candidate genes for the transcriptional regulation of *CESA* gene expression have been successfully identified using the same type of analysis in various species (Brown et al., 2005; Persson et al., 2005; Yamaguchi et al., 2010; Ruprecht et al., 2011; Handakumbura et al., 2018). Among the Bay-0/Sha RILs, the expression of *CESA4, 7*, and *8* were significantly correlated as were the primary wall *CESA*s (Table 1).

**Table 1:** Pairwise Pearson’s correlation coefficients of means of relative gene expression of the Bay-0/Sha recombinant inbred line population.

Transcript	*CESA4*	*CESA7*	*CESA8*	*CESA1*	*CESA3*	*CESA6*	*CESA9*	*CESA2*	*CESA5*
*CESA4*	–	–	–	–	–	–	–	–	–
*CESA7*	0.35	–	–	–	–	–	–	–	–
*CESA8*	0.35	0.32	–	–	–	–	–	–	–
*CESA1*	0.46	0.22	0.23	–	–	–	–	–	–
*CESA3*	0.44	0.22	0.23	0.80	–	–	–	–	–
*CESA6*	0.41	0.22	0.26	0.68	0.76	–	–	–	–
*CESA9*	0.14	0.19	0.02	0.12	0.2	0.14	–	–	–
*CESA2*	0.35	ns	0.28	0.57	0.73	0.50	0.17	–	–
*CESA5*	0.35	0.23	0.24	0.70	0.75	0.58	0.14	0.61	–
*CESA10*	ns	0.19	ns	ns	ns	ns	0.11	ns	ns

*All values are significant at P < 0.01 unless identified as not significant (ns).*

### Secondary Wall *CESA* eQTL

Among the genes whose expression was measured using the ATH1 microarray, 69% were associated with an eQTL and each transcript was mapped to an average of 2.34 loci (West et al., 2007). To pinpoint the cause of *CESA* gene expression variation among RILs, we searched for eQTL for these genes (Figure 1B). An eQTL for each of the *CESA*s was mapped to a position of the genome outside of those genes, i.e., *trans*-eQTL (Figure 1B and Table 2). A *trans*-eQTL common to all three secondary wall *CESA* genes was found near the bottom of chromosome 1. Overlapping *trans*-eQTLs were identified for *CESA4* and *8* on chromosomes 2 and 4. An eQTL unique to *CESA4* with the greatest LOD score of 17.95 was found on chromosome 5, coincident with the *CESA4* genomic locus. *CESA4* transcript abundance in RILs varied significantly depending on the presence of the Bay-0 *CESA4* promoter allele (*CESA4^Bay^*) or the Sha *CESA4* promoter allele (*CESA4^Sha^*) (Figure 1C). No differences were observed for the expression of other secondary wall *CESA* genes, *CESA7* or *CESA8*, between RILs with either *CESA4^Bay^* or *CESA4^Sha^* (Figure 1C).

**Table 2:** Summary of *CESA* eQTL.

Chromosome	Map position (cM)	Peak (bp)	Gene	eQTL type
1	69.5–74.5	24,374,008	*CESA1*	*Trans*
1	76.7–87.52	27,088,847	*CESA1*	*Trans*
1	85.5–100.9	29,016,886	*CESA7*	*Trans*
1	92.4–99.9	29,016,886	*CESA4*	*Trans*
1	92.4–100.9	29,016,886	*CESA8*	*Trans*
2	24.5–41.4	11,095,452	*CESA9*	*Cis*
2	38.5–55.9	13,192,607	*CESA2*	*Trans*
2	38.5–55.9	13,192,607	*CESA5*	*Trans*
2	39.5–47.9	13,192,607	*CESA8*	*Trans*
2	42.4–47.9	13,192,607	*CESA3*	*Trans*
2	43.4	13,192,607	*CESA4*	*Trans*
3	0.0–4.2	786,303	*CESA5*	*Trans*
4	28.7–54.6	11,524,362	*CESA8*	*Cis*
4	52.6–64.9	15,790,523	*CESA4*	*Trans*
4	57.4–63.9	17,261,718	*CESA1*	*Cis*
5	56.5–78.5	18,662,765	*CESA4*	*Cis*
5	68.7–86.5	23,879,425	*CESA5*	*Trans*
5	73.5–96.9	25,924,795	*CESA6*	*Cis*

*Position was calculated using the global 5% significance threshold 2.616 (West et al., 2007).*

### A SNP in the *CESA4* Promoter at the *CESA4 Cis*-eQTL Induces Differential Binding of Cell Wall Thickening NAC Regulators

**FIGURE 2:**
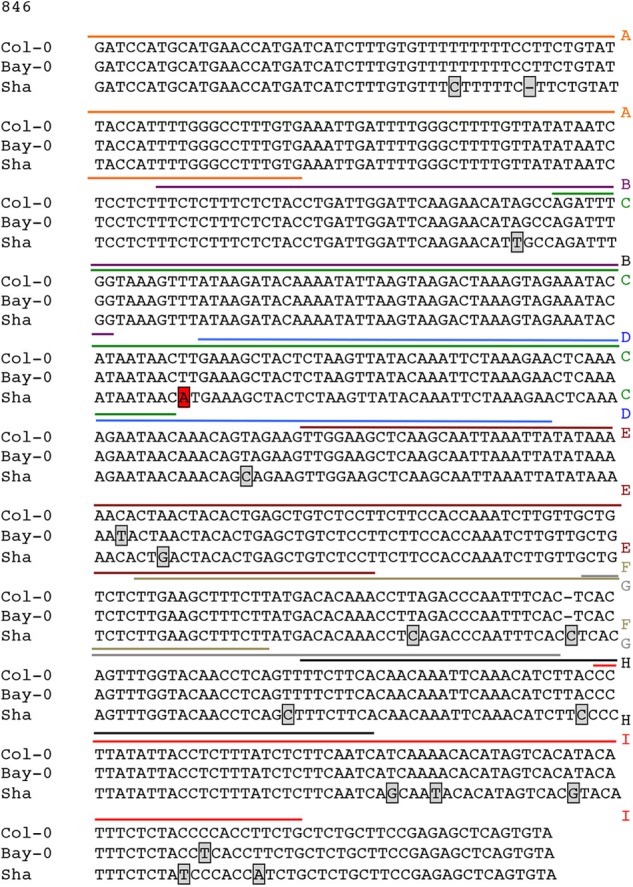
Sequence analysis of promoter of *CESA4* for Col-0, Bay-0, and Sha accessions. Sequencing and alignment of *CESA4* promoter contains only one single nucleotide polymorphism between *CESA4^Bay^* and *CESA4^Col^* and sixteen SNPs between *CESA4^Sha^* and *CESA4^Col^*. Colored lines and letter above the sequence indicate different fragments tested for affinity with NAC proteins. Boxes indicate sequence polymorphisms.

**FIGURE 3:**
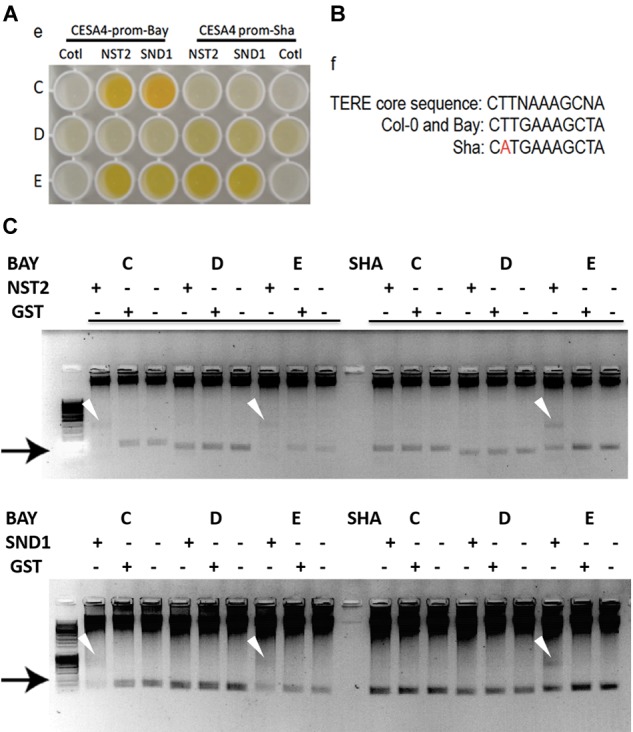
Yeast one-hybrid and protein–DNA interactions within *CESA4* promoter. **(A)** Nine overlapping fragments (A–I) of *CESA4^Bay^* and *CESA4^Sha^* promoters were screened by yeast one-hybrid with *CESA4^Col^* promoter interacting proteins. SND1 and NST2 interacted with two *CESA4^Bay^* fragments (C and E) but only a single *CESA4^Sha^* fragment (E). **(B)** A single nucleotide polymorphism disrupts the second position of a perfect TERE motif in *CESA4^Sha^* fragment C. **(C)** Electrophoretic mobility shift assay for interactions between *CESA4^Bay^* and *CESA4^Sha^* promoters and NST2 and SND1. This polymorphic fragment *CESA4^Bay^* fragment C failed to interact with SND1 and NST2.

Possible mechanisms for such a *cis*-eQTL include functional polymorphisms in the promoter of the gene in question, and we hypothesized that this change may disrupt interaction of SND1 or NST2, which we previously identified to interact with the *CESA4* promoter using yeast one-hybrid (Taylor-Teeples et al., 2015). As such, we sequenced the promoters of Bay-0, Sha, and Col-0 and identified 16 single nucleotide polymorphisms (SNPs) between *CESA4^Sha^* and *CESA4^Col^* and only two SNPs between *CESA4^Bay^* and *CESA4^Col^* (Figure 2). To specify which of the 16 SNPs were likely responsible for the *CESA4 cis*-eQTL, we screened nine overlapping fragments (A–I) of *CESA4^Bay^* and *CESA4^Sha^* promoters by yeast one-hybrid with *CESA4^Col^* promoter interacting proteins (Figures 2, 3A). In this assay, transcription factor proteins were fused to the Gal4 activation domain and each protein is tested for an interaction with the *CESA4* promoter fragments immediately upstream of the *lacZ* reporter. A positive interaction can result in the cleavage from colorless ortho-nitrophenyl-β -D-galactoside by β-galactosidase resulting in a yellow color. The well-characterized wall thickening regulators SND1 and NST2 interacted with two *CESA4^Bay^* fragments (C and E) but only a single *CESA4^Sha^* fragment (E). One SNP in this region disrupts the second position of a perfect *TERE* motif in *CESA4^Sha^* fragment C (Figure 3B). This polymorphic fragment failed to interact with SND1 and NST2 (Figure 3A). This suggests the expression differences between *CESA4^Bay^* and *CESA4^Sha^* could have occurred as a consequence of differential binding of NST2 or SND1 proteins to the *TERE* motif in fragment C.

To further explore the possibility of this regulatory mechanism, EMSA was performed to confirm the differential protein-DNA with probes corresponding to *CESA4* promoter fragments C, D, and E in the presence or absence of extracts of *Escherichia coli* expressing GST-NST2 and GST-SND1 (Figure 3C). As anticipated, differences in mobility were observed with the *TERE* motif-containing fragment C of *CESA4^Bay^* but not with the corresponding fragment of *CESA4^Sha^*, confirming our yeast one-hybrid observations. Also consistent with our yeast one-hybrid results, fragment D from both accessions did not produce a DNA species with retarded mobility, but the TERE motif-containing fragment E did in both cases. Bacterial extracts harboring the empty GST vector did not produce any comparable shifted species. Taken together, these data suggest that the *CESA4^Sha^ TERE* motif polymorphism may result in diminished binding by SND1 and NST2 proteins.

### *CESA4* Is Differentially Expressed in *CESA4^Bay^* and *CESA4^Sha^* Near Isogenic Lines

**FIGURE 4:**
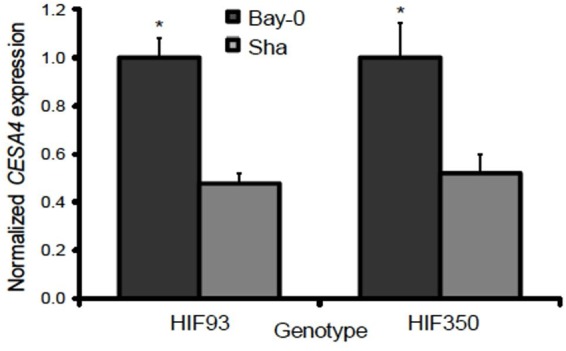
Normalized gene expression results from qPCR between heterogeneous inbred families (HIFs) segregating for parental Bay-0 and Sha alleles in *CESA4* promoter region demonstrated differential binding. Transcript abundance in developing stems containing *CESA4^Bay^* was twofold greater than that of *CESA4^Sha^* in both pairs of NILs. ^∗^*P* < 0.01.

Resolving the effects of the *CESA4^Bay^* and *CESA4^Sha^ cis*-regulatory region is confounded by the entirety of the sequence variation between Bay-0 and Sha, which are maintained in different combinations among the RILs. To isolate the influence of the *CESA4* locus, we identified RILs with residual heterozygosity at *CESA4* and tested derived NILs. We isolated the NILs from heterozygous inbred family 93 (HIF93) and HIF350, segregating for the *CESA4* promoter interval that demonstrated differential binding. Transcript abundance in developing stems containing *CESA4^Bay^* was twofold greater than that of *CESA4^Sha^* in both pairs of NILs (Figure 4). These results further support the differential binding between Bay-0 and Sha by NST2 and SND1, thus changing expression of *CESA4*.

### No Differences in Cell Wall Properties Were Observed Between *CESA4^Bay^* and *CESA4^Sha^* Near Isogenic Lines

**FIGURE 5:**
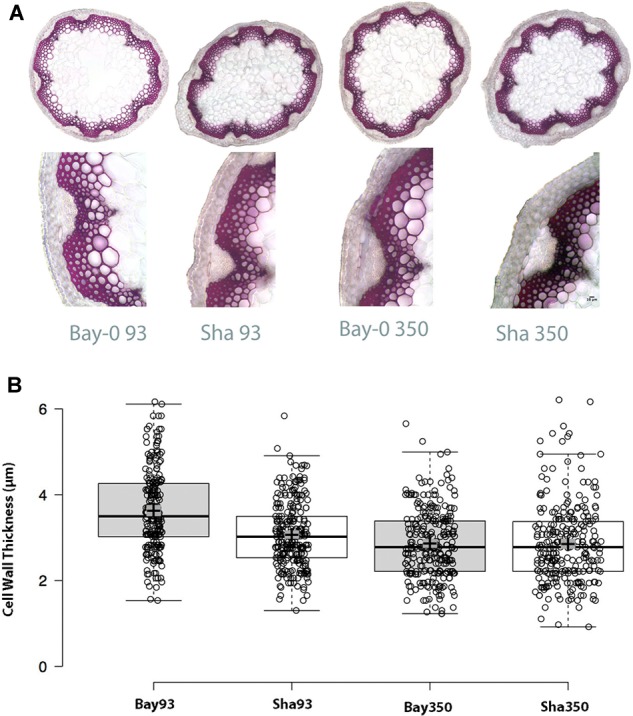
**(A)** Phloroglucinol stained cross sections with representative focus areas for measurements. **(B)** Cell wall thickness variation between Bay-0 and Sha NILS. *CESA4^Bay^* NIL93 was the only line to exhibit slightly thicker cell walls, while the other three lines were similar. Center lines show the medians; box limits indicate the 25th and 75th percentiles as determined by R software; whiskers extend 1.5 times the interquartile range from the 25th and 75th percentiles; crosses represent sample means with surrounding gray box indicating 95% confidence interval.

Considering the critical role of CESA4 in plant structure, we wished to evaluate the phenotypic effect of the *cis-*eQTL. Bay-0 and Sha *CESA4* NILs were indistinguishable at the macro level, but a subtle consequence at the cellular level remained a possibility. To explore the effect of the *CESA4 cis-*eQTL on cellulose, Bay-0 and Sha were first examined with stem histology to measure cell wall thickness. Stem cross sections were indistinguishable between NILs (Figure 5A). All xylem and interfascicular cells had well-defined edges and equivalent phloroglucinol staining. The *CESA4^Bay^* NIL93 exhibited an average cell wall thickness of 3.57 μm, which was slightly greater than the 3.07 μm thickness of their *CESA4^Sha^* NIL93 counterparts. The *CESA4^Bay^* NIL93 samples were quite variable with a standard deviation of 0.45 μm. Cell wall thickness of *CESA4^Bay^* and *CESA4^Sha^* NIL350 were almost identical, 2.86 and 2.85 μm, respectively (Figure 5B).

**FIGURE 6:**
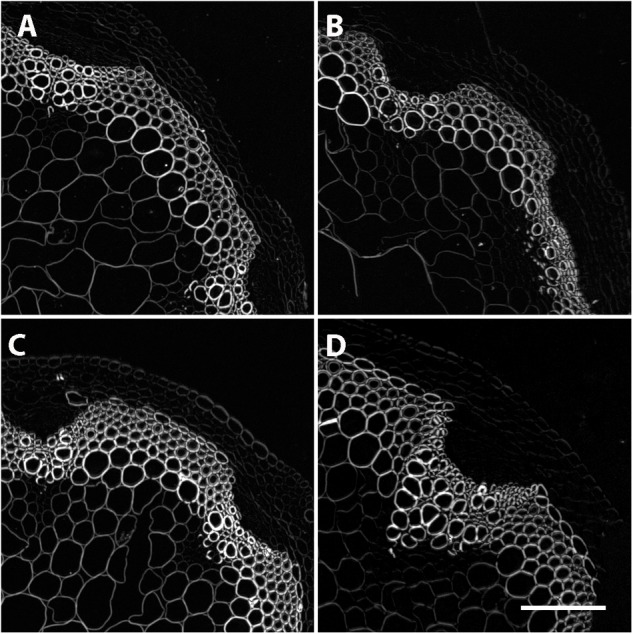
Polarized light microscopy on stem cross sections revealed no differences in crystallinity. Representative images of **(A)** Sha 93, **(B)** Bay-0 93, **(C)** Sha 350, and **(D)** Bay-0 350.

While no significant differences in overall lignin staining or cell thickness was observed, we hypothesized that the reduction in *CESA4* expression may still compromise the cellulose crystalline structure of the NILs carrying the *CESA4^Sha^* allele. To evaluate this, we employed Updegraff assay to quantify crystalline cellulose and polarized light microscopy as a secondary indicator of crystallinity. Percent composition of crystalline cellulose was slightly higher in *CESA4^Bay^* NIL93 than *CESA4^Sha^* NIL93, 28.2% vs. 26.8%, but lower in *CESA4^Bay^* NIL350 that *CESA4^Sha^* NIL350, 27.0% vs. 29.2%. Percent cellulose crystallinity was marginally greater in the Bay-0 parental accession than Sha, 27.0% vs. 25.3% (Supplementary Figure 2). However, the NILs were not significantly different. Polarized light images of all samples mirrored brightfield images and were indistinguishable between NILs (Figure 6). Birefringent retardance was strong and consistent in xylem and interfascicular cells, revealing no difference in quantities or order of crystalline cellulose. The comparable phenotypes between NILs revealed in both the histological and chemical assays suggest the decreased *CESA4* expression observed in *CESA4^Sha^* NILs does not disrupt cellulose abundance or crystallinity.

### The Secondary Wall Associated NAC Proteins Bind the Same Sequence

**FIGURE 7:**
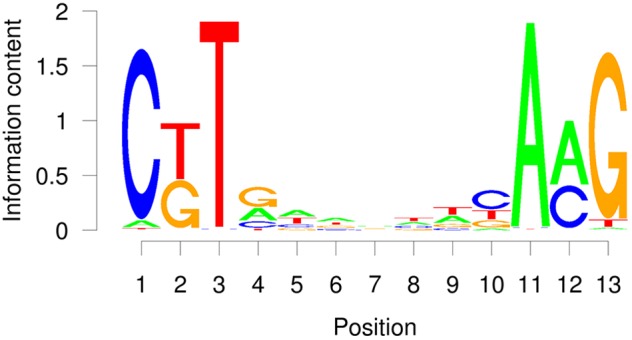
Average probability matrix motif for the VND, NST, and SND secondary cell wall NAC protein DAP-seq showing the probability of a nucleotide at each position.

The TERE and SNBE are compatible sequences, independently identified as binding sites of VND and NST NAC proteins (Pyo et al., 2007; Ohashi-Ito et al., 2010; Zhong et al., 2010). The TERE sequence, CTTNAAAGCNA, is consistent with the following internal sequence of the SNBE: CTTNNNNNNNA. To further resolve this binding site we explored the DNA affinity purification sequencing (DAP-seq) data previously generated for *A. thaliana* transcription factors and Col-0 genomic DNA (O’Malley et al., 2016). We searched the binding peaks of DAP-seq data for available VND, SND, and NST proteins: VND1, 2, 3, 4, 6, SND2, SND3, and NST1. The data included DNA sequence from libraries of DNA where methylcytosines were removed by PCR and unamplified libraries. There is a striking similarity between the top enriched motif described by the DAP-seq and the SNBE/TERE motif (Supplementary Table 3). The core DAP-seq derived motif similarity across all three groups of NACs is three positions flanking seven nucleotides of any sequence: C(G/T)TNNNNNNNA(A/C)G. We refer to this as the VND/NST/SND element (VNS, Figure 7). Interestingly, the motif appears to be palindromic depending on the variable positions. We searched the sequences of the binding sites and found the prefix CTT and AAG suffix to represent more than half of the total occurrences of the VNS, however, each possible sequence was similar to the frequency in the genome (Table 3). Therefore, there does not appear to be a preference for the variable positions. There was a low frequency of an A or a T in the first and last position of the binding site, respectively. The NAC binding site in the *CESA4* promoter is consistent with both the TERE and a VNS, but with a T at the last position.

**Table 3:** Comparison of VND/NST/SND (VNS) NAC protein binding motif variants by percentage in the *Arabidopsis thaliana* genome.

VNS element variants	VNS sites in the Col-0 genome sequence	VND/NST/SND DAP-seq VNS peak motifs
	**%**	

CTTNNNNNNNAAG	57.0	55.9
CTTNNNNNNNACG	18.0	23.4
CGTNNNNNNNAAG	18.0	23.4
CGTNNNNNNNACG	8.5	11.5

## Discussion

We investigated the variable expression of *CESA4* in a RIL population of *A. thaliana*. After observation of an eQTL at the *CESA4* locus, SNP analysis and transcription factor binding assays revealed a disruption in an otherwise perfect TERE motif in the *CESA4^Sha^* allele. Chemical and histological analysis of the cell wall composition was unable to detect any differences between NILs carrying the *CESA4^Bay^* or *CESA4^Sha^* allele.

Natural variation in transcript abundance and its association with growth and biomass traits has been documented in a number of instances. Analysis of transcript abundance in a *Eucalyptus* backcross population revealed downregulation of lignin biosynthesis genes is associated with an increase in growth rate (Kirst et al., 2004). Meanwhile, upregulation of genes may confer either positive or negative effects on biomass accumulation; eQTL analysis of high and low biomass pools of *Poplar* revealed that of the identified loci of differential expression, half were upregulated in high biomass trees and half were upregulated in low biomass trees (Du et al., 2015). Alternatively, genotypic variation of patterns of differential expression between mature and immature tissues can also be an indicator of advantageous biomass traits, as presented in a recent study of the sugarcane transcriptome, in which *ShCESA4* and *ShCESA7* were differentially expressed between top and bottom internodes in high fiber genotypes only (Kasirajan et al., 2018). Notably, increased expression of the *CESAs* is frequently associated with higher sugar content or biomass. In alfalfa, *CESA4* was upregulated in genotypes exhibiting greater cellulose content, and similarly, upregulation of *CESA4* in shrub willow was associated with increased total polysaccharide content (Yang et al., 2010; Serapiglia et al., 2012). Though the present study concurs with others that underline the occurrence of naturally variable expression patterns, it differs with the lack of an association between *CESA4* expression and cellulose content. This study also diverges from previous reports of *CESA* expression, as the correlation coefficients among the secondary cell wall *CESAs* were relatively low (Appenzeller et al., 2004). This discrepancy is likely due to the low enrichment of secondary cell walls in the tissue tested.

Variation in transcript abundance among genotypes may be caused by non-synonymous or synonymous SNPs in coding regions, as well as SNPs in introns and 3′ and 5′UTR, as was reported in *Pinus taeda, Eucalyptus*, and *Picea glauca* (Kirst et al., 2004; Beaulieu et al., 2011; Palle et al., 2013). Such SNPs can induce either *trans*-eQTLs, functioning by affecting the expression of transcription factor targets or other regulatory interactions, or *cis*-eQTLs, modulating expression by discrepancies in promoter sequence, as presented here. Though non-coding regions face less selective pressure than coding sequences, development-specific expression profiles and regions responsible for transcription factor binding have been shown to have greater conservation of motifs in promoters across species (Creux et al., 2008; Ding et al., 2012). We describe a loss-of-function mutation to the *TERE* motif, complementing previous reports of the effects of aberrations to this 11 bp sequence (Pyo et al., 2007).

A number of studies have investigated the effects of perturbed *CESA* expression on biomass production, and several studies have underlined the flexibility of species to tolerate moderate discrepancies in expression. amiRNA inhibition of *CESA4* expression in *B. distachyon*, in which *BdCESA4* expression was reduced almost 10-fold, resulted in compromised cellulose production, yet the same study found that reduction in *CESA7* expression by only 1.5-fold resulted in only moderate effects on cell wall composition (Handakumbura et al., 2013; Mazarei et al., 2018). A similar study of *Panicum virgatum* highlighted that modest reductions in expression caused no detectable changes to plant structure or biomass production, and only samples with a >40% reduction in expression were compromised in cell wall traits (Mazarei et al., 2018). Notably, studies reporting the most profound phenotypes typically discuss mutations in the coding region of genes or complete loss-of-function alleles (Tanaka et al., 2003; Taylor et al., 2003; Joshi et al., 2011).

The presence of loss-of-function *cis-*eQTLs across viable accessions found in variable geographic locations also underlines the ability of the plant genome to tolerate expression irregularities and perhaps poses more questions about posttranscriptional and translational regulation, as well as the role of local environment in trait variance (Vuylsteke and van Eeuwijk, 2008; Zan et al., 2016). The results suggest that regulatory mechanisms may be at play, highlighting the elasticity of the plant genome and proteome. A plethora of both naturally occurring and mutagenesis-induced transcript discrepancies have been associated with a structural or developmental phenotype, and the same was expected in this study. However, resistance to cellulose perturbation has also been reported. For example, co-suppression of several *CESAs* in barley (*Hordeum vulgare*) caused by constitutive expression using the CaMV35S promoter was only sometimes (25% of the time) associated with a compromised cell wall phenotype (Tan et al., 2015). Transgenic overexpression of each secondary cell wall *CESA, CESA4, 7*, and *8*, resulted in lower transcript levels for all three endogenous secondary cell wall *CESAs*. Moreover, transcript levels of all three *CESAs* tended to correlate, regardless of which *CESA* cDNA was driven by the CaMV35S promoter, suggesting that regulatory mechanisms limited by synthase complex stoichiometry are at play (Tan et al., 2015).

Co-regulation of the CSC subunits at the transcript level was not reported in HIFs for this study, but posttranscriptional regulation poses another possible mechanism to explain the lack of phenotypic effect. While transcript analysis is a powerful tool and can provide invaluable data on genome regulation, stress response, and more, mRNA accumulation alone is not enough to draw definitive conclusions about protein expression. Indeed, the global correlation between the transcriptome and proteome in both prokaryotes and eukaryotes has been found to be weak, at best (Gygi et al., 1999; Washburn et al., 2003; Soto-Suárez et al., 2016). Posttranscriptional regulation mechanisms, half-life, and localization and interactions all may play a role in protein expression levels, causing them to differ from transcript abundance.

A number of studies have identified cases where protein expression of complex subunits is posttranscriptionally controlled and seemingly limited by complex stoichiometry; generally, mRNA levels of individual subunits have not been found to correlate with complex expression (Washburn et al., 2003; Hajduch et al., 2010; Lalanne et al., 2018). For example, it was found that the alpha and beta plastidial pyruvate kinase subunits had similar protein expression but discrepant transcript expression, suggesting that the requirements of complex assembly can be a regulatory factor in protein accumulation. A similar scenario could likely be occurring in the CSC, and further studies would benefit from an investigation of CESA protein expression levels.

## Author Contributions

JO, LL, and SH conceived and designed the study. JO, LL, and GT acquired the data. JO, LL, SL, KM, and SH analyzed and interpreted the data. JO, LL, and SH drafted the manuscript.

## Conflict of Interest Statement

The authors declare that the research was conducted in the absence of any commercial or financial relationships that could be construed as a potential conflict of interest.
