# Supplementary material for: Secondary Wall Regulating NACs Differentially Bind at the Promoter at a CELLULOSE SYNTHASE A4 Cis-eQTL
Source: Front Plant Sci. 2018 Dec 21;9:1895. doi: 10.3389/fpls.2018.01895 (PMC6309453; doi:10.3389/fpls.2018.01895)
Supplement: Supplementary file 4 [file Data_Sheet_1.docx]

Supplementary Material

**Secondary wall regulating NACs differentially bind at the promoter at a *CELLULOSE SYNTHASE A4* *cis*-eQTL**

**Jennifer Olins^1,†^, Li Lin^1,†^, Scott J. Lee^1,2^, Gina M. Trabucco^1,3^, Kirk J.-M.**

**MacKinnon^1,3^, Samuel P. Hazen^1,*^**

*** Correspondence:** Samuel Hazen: hazen@bio.umass.edu

**
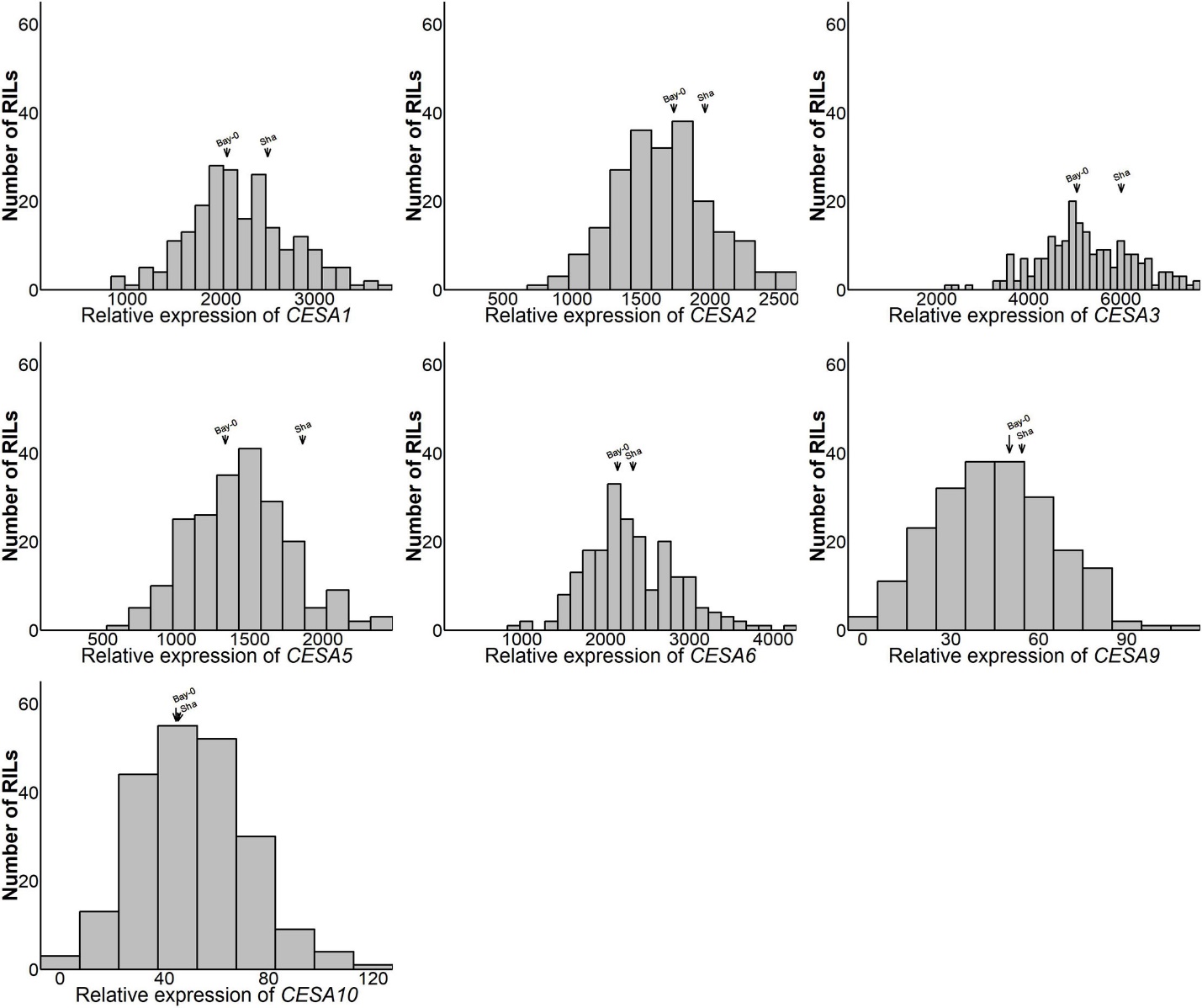
**

**Supplementary Figure 1.** Histograms of relative expression of *CESA1/2/3/5/6/9/10* within recombinant inbred line population (Bay-0 x Sha).


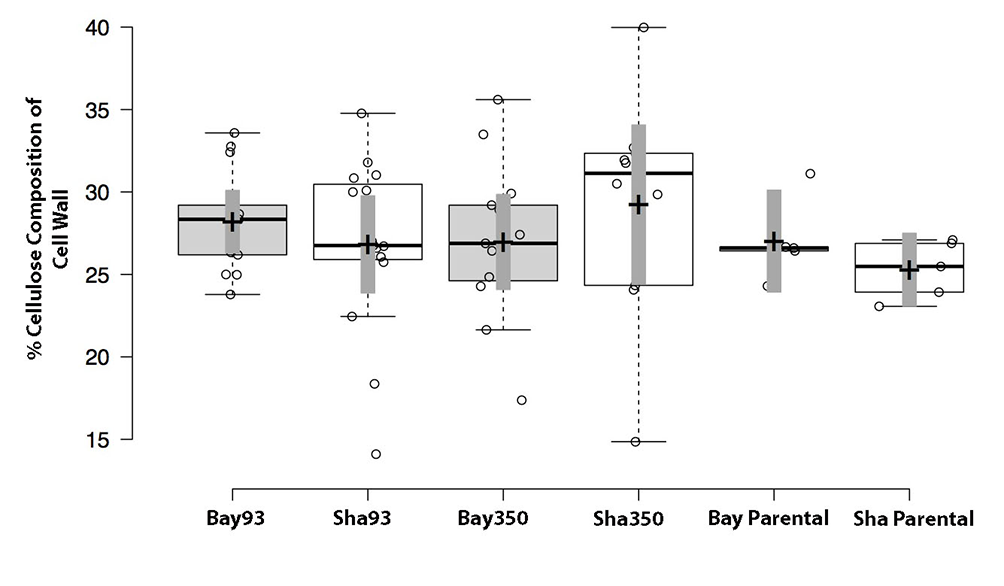


**Supplementary Figure 2. Percent cell wall composition of crystalline cellulose between Bay-0 and Sha NILs and parental lines.** Cellulose composition was slightly higher in *CESA4^Bay^* than *CESA4^Sha^* NIL93 and parental lines, but lower in NIL350. Center lines show the medians; box limits indicate the 25th and 75th percentiles as determined by R software; whiskers extend 1.5 times the interquartile range from the 25th and 75th percentiles; crosses represent sample means with surrounding grey box indicating 95% confidence interval.
